# Supplementary figures and images for: Determining the Efficacy, Feasibility, and Impact of Storage Conditions on At-Home Blood Collection Kits for Proteomic Studies
Source: medRxiv. 2025 May 16:2025.05.14.25327396. Preprint. [Version 1] doi: 10.1101/2025.05.14.25327396 (PMC12132123; doi:10.1101/2025.05.14.25327396)

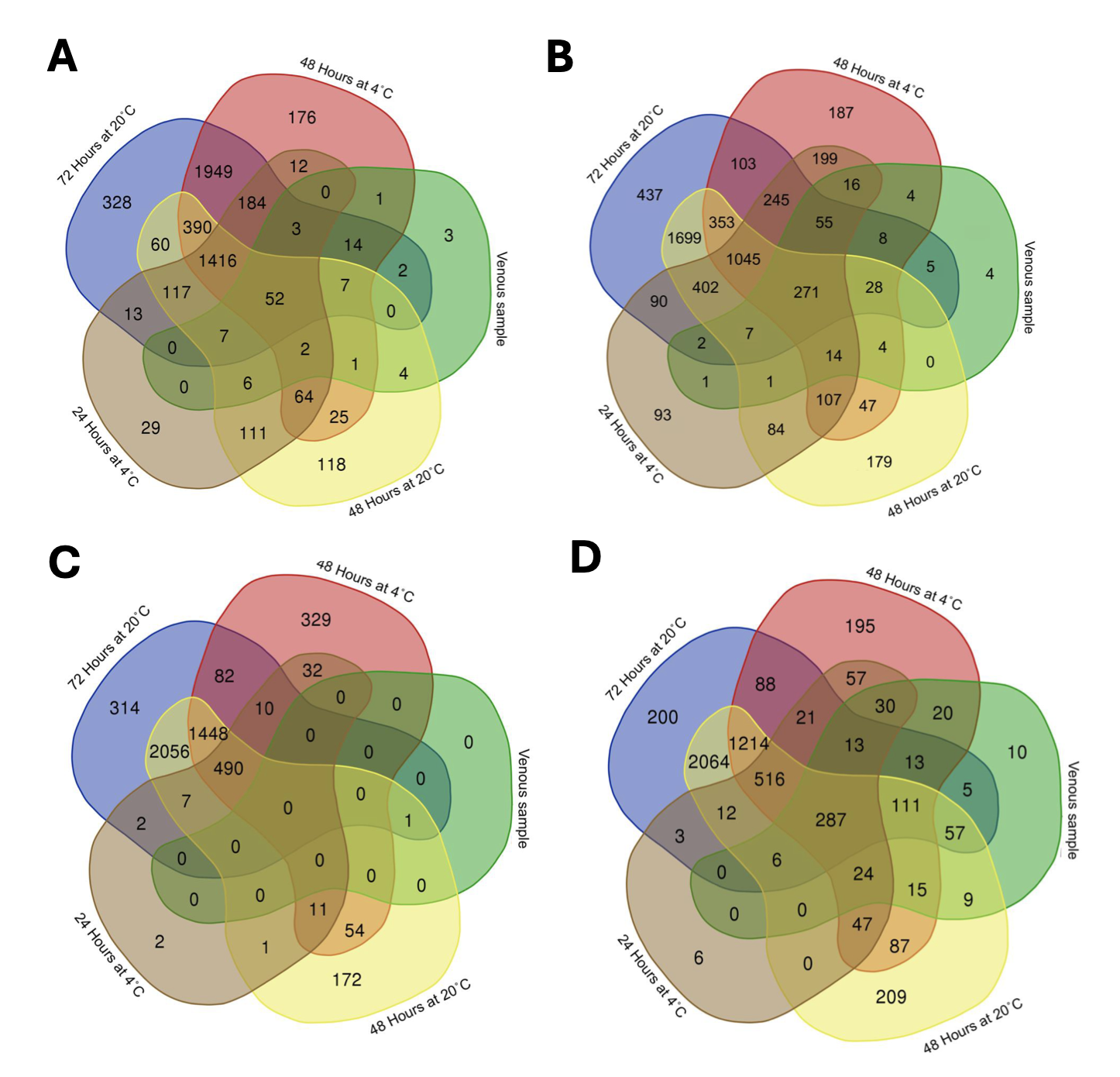

Supplement: Supplement 1 — Supplementary Table S1. Proteins with significantly altered detection for all samples Supplementary Table S2. Proteins with significantly altered detection in every sample Supplementary Table S3. Proteins with significantly altered detection in no samples Supplementary Table S4. PERMANOVA and Kruskal-Wallis test results Supplementary Table S5. Proteins with significantly altered detection in Model B Supplementary Table S6. Proteins with significantly altered detection in Model C (Temperature) Supplementary Table S7. Proteins with significantly altered detection in Model C (Time) Supplementary Figure S1. SomaLogic Quality Statement Quality statement provided by SomaLogic, denoting the quality of the samples on arrival and other information regarding quality, calibration, and the assay. Supplementary Figure S2. Venn diagrams for individual variation Venn Diagrams for each participant (A through D, in panels A through D) which show the overlap in proteins which were detected at significantly different levels in each sample, compared to the capillary baseline samples. Supplementary Figure S3. Venn diagram of proteins altered in all samples per individual Venn diagram of the significantly impacted proteins in all of participant A, B, and D’s samples (participant C had no proteins altered in all samples compared to the baseline). Supplementary Figure S4. Significantly changed protein levels between capillary and venous samples of individual participants Four-panel bar graph of the proteins with significantly impacted detection for each of the participants when comparing the venous-collected sample to the capillary baseline. Four panels (A through D) correspond to participants A through D. Box-and-whisker plots are overlayed on the bars to show the spread of protein detection for each KEGG subcategory. [file media-1.zip › Supplemental_Docs+Figures/Supplementary Figure S2.tiff]

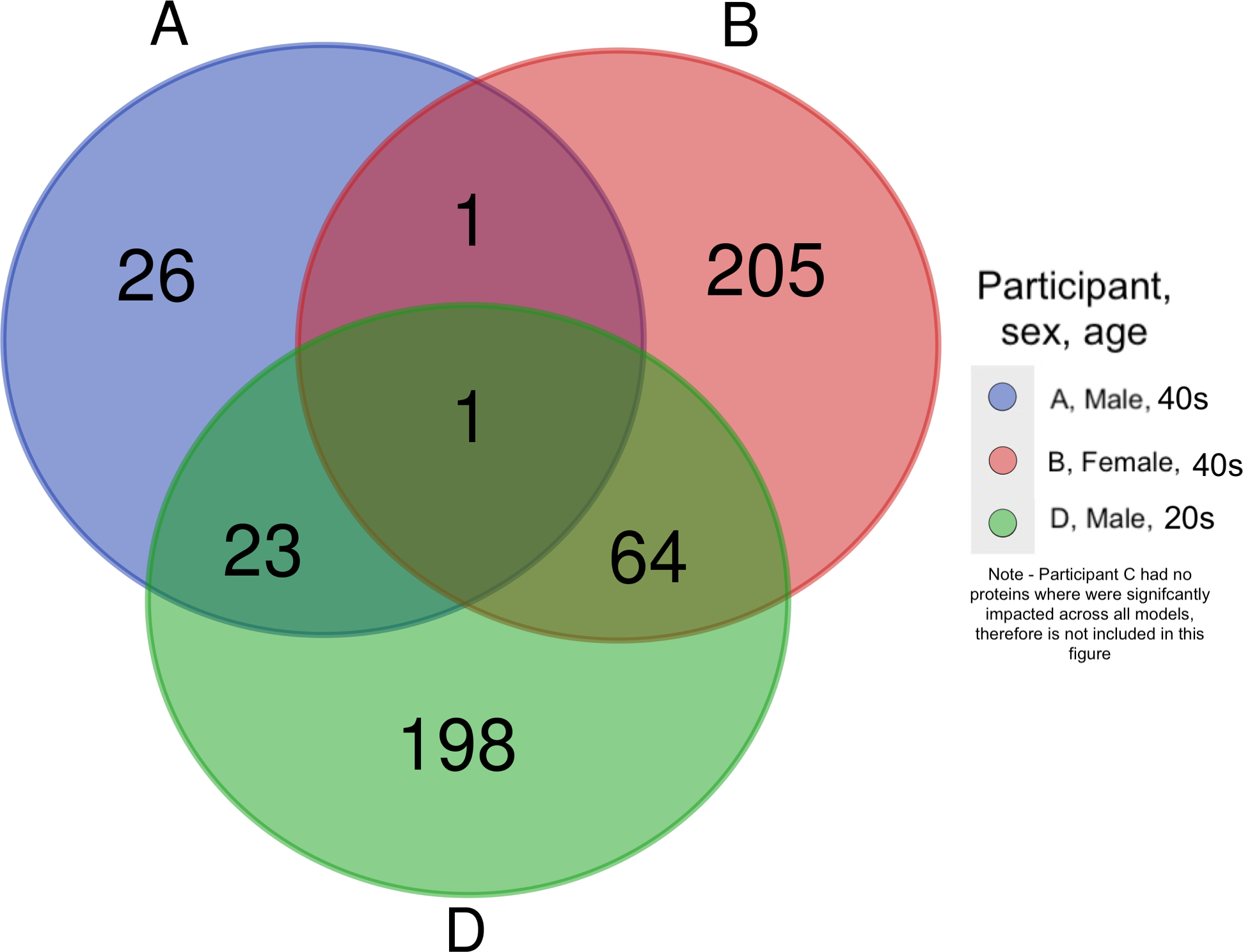

Supplement: Supplement 1 — Supplementary Table S1. Proteins with significantly altered detection for all samples Supplementary Table S2. Proteins with significantly altered detection in every sample Supplementary Table S3. Proteins with significantly altered detection in no samples Supplementary Table S4. PERMANOVA and Kruskal-Wallis test results Supplementary Table S5. Proteins with significantly altered detection in Model B Supplementary Table S6. Proteins with significantly altered detection in Model C (Temperature) Supplementary Table S7. Proteins with significantly altered detection in Model C (Time) Supplementary Figure S1. SomaLogic Quality Statement Quality statement provided by SomaLogic, denoting the quality of the samples on arrival and other information regarding quality, calibration, and the assay. Supplementary Figure S2. Venn diagrams for individual variation Venn Diagrams for each participant (A through D, in panels A through D) which show the overlap in proteins which were detected at significantly different levels in each sample, compared to the capillary baseline samples. Supplementary Figure S3. Venn diagram of proteins altered in all samples per individual Venn diagram of the significantly impacted proteins in all of participant A, B, and D’s samples (participant C had no proteins altered in all samples compared to the baseline). Supplementary Figure S4. Significantly changed protein levels between capillary and venous samples of individual participants Four-panel bar graph of the proteins with significantly impacted detection for each of the participants when comparing the venous-collected sample to the capillary baseline. Four panels (A through D) correspond to participants A through D. Box-and-whisker plots are overlayed on the bars to show the spread of protein detection for each KEGG subcategory. [file media-1.zip › Supplemental_Docs+Figures/Supplementary Figure S3.tif]

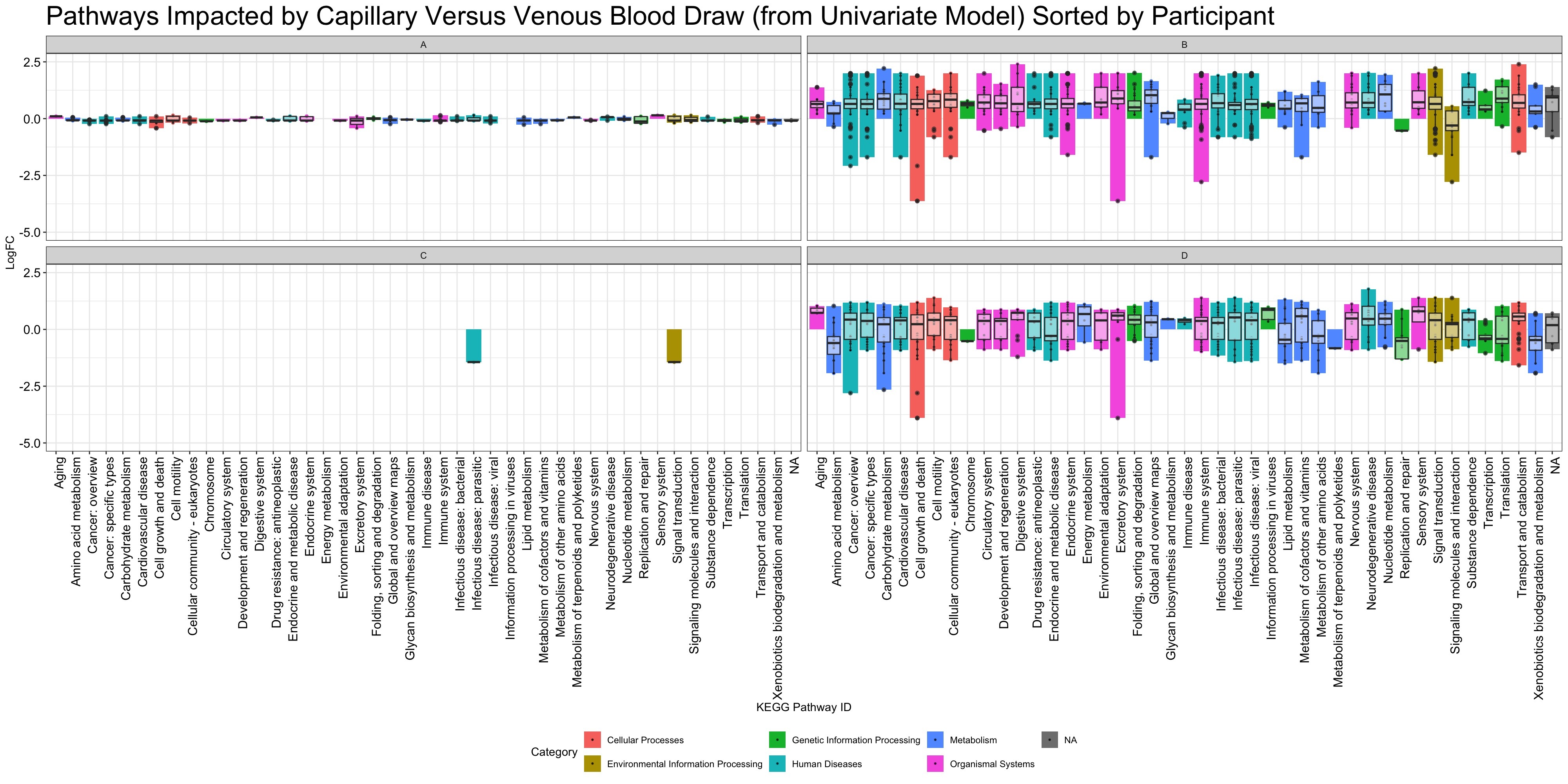

Supplement: Supplement 1 — Supplementary Table S1. Proteins with significantly altered detection for all samples Supplementary Table S2. Proteins with significantly altered detection in every sample Supplementary Table S3. Proteins with significantly altered detection in no samples Supplementary Table S4. PERMANOVA and Kruskal-Wallis test results Supplementary Table S5. Proteins with significantly altered detection in Model B Supplementary Table S6. Proteins with significantly altered detection in Model C (Temperature) Supplementary Table S7. Proteins with significantly altered detection in Model C (Time) Supplementary Figure S1. SomaLogic Quality Statement Quality statement provided by SomaLogic, denoting the quality of the samples on arrival and other information regarding quality, calibration, and the assay. Supplementary Figure S2. Venn diagrams for individual variation Venn Diagrams for each participant (A through D, in panels A through D) which show the overlap in proteins which were detected at significantly different levels in each sample, compared to the capillary baseline samples. Supplementary Figure S3. Venn diagram of proteins altered in all samples per individual Venn diagram of the significantly impacted proteins in all of participant A, B, and D’s samples (participant C had no proteins altered in all samples compared to the baseline). Supplementary Figure S4. Significantly changed protein levels between capillary and venous samples of individual participants Four-panel bar graph of the proteins with significantly impacted detection for each of the participants when comparing the venous-collected sample to the capillary baseline. Four panels (A through D) correspond to participants A through D. Box-and-whisker plots are overlayed on the bars to show the spread of protein detection for each KEGG subcategory. [file media-1.zip › Supplemental_Docs+Figures/Supplementary Figure S4.tiff]
